# Whole-Genome-Sequence-Based Characterization of Extensively Drug-Resistant Acinetobacter baumannii Hospital Outbreak

**DOI:** 10.1128/mSphere.00934-19

**Published:** 2020-01-15

**Authors:** Ghiwa Makke, Ibrahim Bitar, Tamara Salloum, Balig Panossian, Sahar Alousi, Harout Arabaghian, Matej Medvecky, Jaroslav Hrabak, Samar Merheb-Ghoussoub, Sima Tokajian

**Affiliations:** a Department of Natural Sciences, Lebanese American University, Byblos, Lebanon; b Department of Microbiology, Faculty of Medicine, University Hospital Pilsen, Charles University, Pilsen, Czech Republic; c Biomedical Center, Faculty of Medicine, Charles University, Pilsen, Czech Republic; d CEITEC VFU, University of Veterinary and Pharmaceutical Sciences Brno, Brno, Czech Republic; e Pierre and Marie Curie University, Paris, France; JMI Laboratories

**Keywords:** *A. baumannii*, CRAB, Tn*2006*, pMAL-1, hospital outbreak

## Abstract

Carbapenem-resistant Acinetobacter baumannii (CRAB) is an important opportunistic pathogen linked to a variety of nosocomial infections and hospital outbreaks worldwide. This study aimed at investigating and characterizing a CRAB outbreak at a large tertiary hospital in Lebanon. A total of 41 isolates were collected and analyzed using pulsed-field gel electrophoresis (PFGE). Whole-genome sequencing (WGS) was performed on all the isolates, and long-read PacBio sequencing was used to generate reference genomes. The multilocus sequence types (MLST), repertoire of resistance genes, and virulence factors were determined from the sequencing data. The plasmid content was analyzed both *in silico* and using the A. baumannii PCR-based replicon typing (AB-PBRT) method. Genome analysis initially revealed two clones, one carrying *bla*_OXA-23_ on Tn*2006* (ST-1305, ST-195, and ST-218) and another carrying *bla*_OXA-72_ on pMAL-1 (ST-502 and ST-2059, a new ST), with the latter having two subclones, as revealed using the Bayesian transmission network. All isolates were extensively drug resistant (XDR). WGS analysis revealed the transmission pathways and demonstrated the diversity of CRAB isolates and mobile genetic elements in this health care setting. Outbreak detection using WGS and immediate implementation of infection control measures contribute to restraining the spread and decreasing mortality.

**IMPORTANCE** Carbapenem-resistant Acinetobacter baumannii (CRAB) has been implicated in hospital outbreaks worldwide. Here, we present a whole-genome-based investigation of an extensively drug-resistant CRAB outbreak rapidly spreading and causing high incidences of mortality at numerous wards of a large tertiary hospital in Lebanon. This is the first study of its kind in the region. Two circulating clones were identified using a combination of molecular typing approaches, short- and long-read sequencing and Bayesian transmission network analysis. One clone carried *bla*_OXA-23_ on Tn*2006* (ST-1305, ST-195, and ST-218), and another carried *bla*_OXA-72_ on a pMAL-1 plasmid (ST-502 and ST-2059, a new ST). A pMAL-2 plasmid was circulating between the two clones. The approaches implemented in this study and the obtained findings facilitate the tracking of outbreak scenarios in Lebanon and the region at large.

## Abstract (precis)

Carbapenem-resistant Acinetobacter baumannii (CRAB) has been implicated in hospital outbreaks worldwide. Here, we present a whole-genome-based investigation of an extensively drug-resistant CRAB outbreak rapidly spreading and causing high incidences of mortality at numerous wards of a large tertiary hospital in Lebanon. This is the first study of its kind in the region. Two circulating clones were identified using a combination of molecular typing approaches, short- and long-read sequencing and Bayesian transmission network analysis. One clone carried *bla*_OXA-23_ on Tn*2006* (ST-1305, ST-195, and ST-218), and another carried *bla*_OXA-72_ on a pMAL-1 plasmid (ST-502 and ST-2059, a new ST). A pMAL-2 plasmid was circulating between the two clones. The approaches implemented in this study and the obtained findings facilitate the tracking of outbreak scenarios in Lebanon and the region at large.

## INTRODUCTION

Acinetobacter baumannii is associated with a broad range of severe wound, skin and soft-tissue, urinary tract, and bloodstream infections, secondary meningitis, and ventilator-associated pneumonia (1). These often result in extended periods of hospitalization and admission to intensive care units (ICUs) (2). A. baumannii survives inside human hosts, on dry surfaces, and on the hands of health care personnel for prolonged periods, and it is often associated with worldwide hospital outbreaks (3, 4).

A. baumannii infections are difficult to treat, having both intrinsic and acquired drug resistance determinants carried on plasmids, transposons, and integrons (5–7). Carbapenem resistance is mostly mediated by oxacillinases (OXAs) and less frequently by metallo-β-lactamases (MBLs) (8). The marked overproduction of class D β-lactamases (CHDLs) is the main mechanism conferring carbapenem resistance in A. baumannii and is caused by intrinsic genes encoding OXA-51-like enzymes and other families of OXA-type CHDLs, including OXA-23-like, OXA-40-like, OXA-58-like, and OXA-143 enzymes (9). The frequency of CHDLs and MBLs in A. baumannii varies in different geographic regions (10).

OXA-51 is the largest group of intrinsic OXA-type β-lactamases identified. It was originally detected in 1996 in A. baumannii recovered from Argentina, and later it became an important marker used for the identification of the organism at the species level (11, 12). IS*Aba1* has been identified in association with different OXA-β-lactamases, including the *bla*_OXA-51-like_ genes. IS*Aba1* is located 7 bp upstream in the opposite direction of *bla*_OXA-51-like_ and provides a promoter that can increase *bla*_OXA-51-like_ gene expression levels by 50-fold (12, 13).

OXA-23 was first identified in A. baumannii strains isolated in the United Kingdom in 1993. Since then, it has been detected worldwide and was linked to the global dissemination of carbapenem-resistant A. baumannii (CRAB) (14–16). Al Atrouni et al. showed that 76.5% of the A. baumannii isolates collected from different hospitals in Lebanon were carbapenem resistant, with the majority (90%) harboring the OXA-23 carbapenemase (17). More recent studies revealed that carbapenem susceptibility among Acinetobacter isolates was 12% (18), and most of the recovered isolates (83%) were susceptible to colistin (19). Moreover, OXA-24 has several variants, including OXA-72. Isolates carrying *bla*_OXA-72_ were associated with a number of hospital outbreaks in Spain, Ecuador, and the United States (13, 16).

In this study, we describe the molecular epidemiology of a CRAB-associated outbreak linked to Tn*2006*- and pMAL-1-mediated clones. The results obtained through whole-genome sequencing (WGS) and single-nucleotide polymorphism (SNP) analysis of 41 isolates collected in 2016 from a hospital in Lebanon were compared to the subtyping patterns obtained by pulsed-field gel electrophoresis (PFGE) and multilocus sequence typing (MLST). WGS data were additionally used to compare the two identified outbreak clones, build transmission networks, explore the genetic relatedness of the isolates causing the outbreak, study their resistomes and virulomes, and determine their circulating mobile genetic elements.

## RESULTS

### Clinical characteristics.

**FIG 1:**
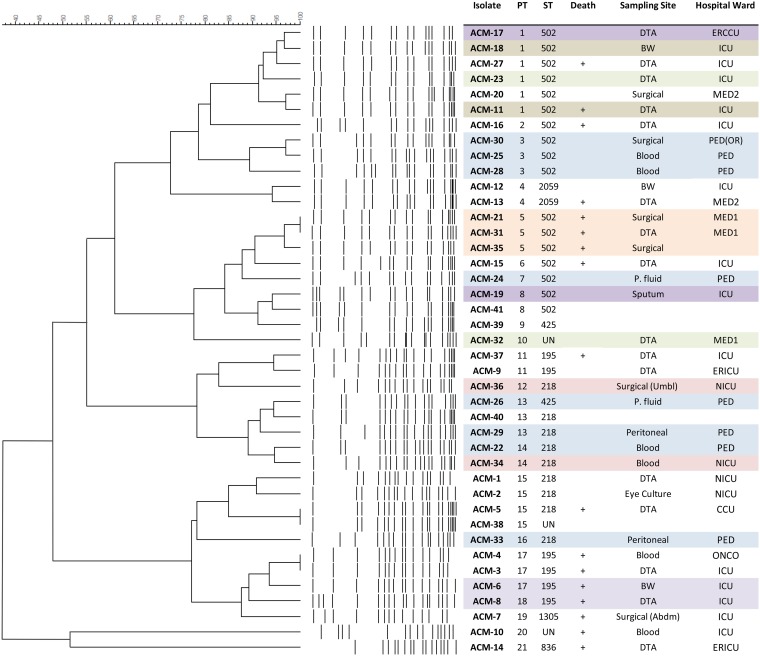
PFGE dendrogram, isolates’ STs, and patients’ information. PT, pulsotype; M, male; F, female; DTA, deep tracheal aspiration; P. fluid, peritoneal fluid; BW, bronchial wash; abdm, abdomen; umbl, umbilical; +, died; ICU, intensive care unit; ERICU, emergency room intensive care unit; CCU, coronary care unit; PED, pediatrics; NICU, neonatal intensive care unit; ONCO, oncology; PED(OR), pediatrics operation room; MED1, medical floor 1; MED2, medical floor 2.

A total of 41 A. baumannii isolates were collected between April and December 2016 from hospitalized patients. Patients’ records were available for 37 of the isolates, designated ACM-1 to -37. These were recovered from 23 different patients with a mean age of 53 ± 27 years, ranging between newborn and 92 years old. Of these, 65% (*n* = 15) were females and 35% (*n* = 8) were males, and more than half of the patients (61%; *n* = 14) died due to different causes, including underlying diseases (Fig. 1; see also Table S1 in the supplemental material).

10.1128/mSphere.00934-19.1 TABLE S1 Patients’ information. M, male; F, female; DTA, deep tracheal aspiration; P fluid, peritoneal fluid; BW, bronchial wash; abdm, abdomen; umbl, umbilical. Classes of antibiotics are in the following colors: yellow, aminoglycosides; blue, β-lactams; green, quinolones; orange, tetracyclines; white, polymyxins; grey, cephalosporin. GM, gentamicin; AK, amikacin; TZP, piperacillin-tazobactam; CAZ, ceftazidime; FEP, cefepime; IMP, imipenem; MEM, meropenem; CIP, ciprofloxacin; LEV, levofloxacin; TMP-SMX, trimethoprim-sulfamethoxazole; COL, colistin. Black indicates resistant, grey indicates intermediate susceptibility, and blank indicates sensitive. −, information not available. MIC, minimal inhibitory concentration in micrograms per milliliter. Download Table S1, PDF file, 0.1 MB. Copyright © 2020 Makke et al. 2020 Makke et al. This content is distributed under the terms of the Creative Commons Attribution 4.0 International license.

Isolates ACM-10 and -14 were found to be A. calcoaceticus and were not part of the outbreak. ACM-14 was used as an outgroup in the phylogenetic analysis. Isolate ACM-26 was a mixed culture (different strains of A. baumannii) recovered from a 3-year-old male patient.

### Antibiotic susceptibility testing and resistance genes.

**FIG 2:**
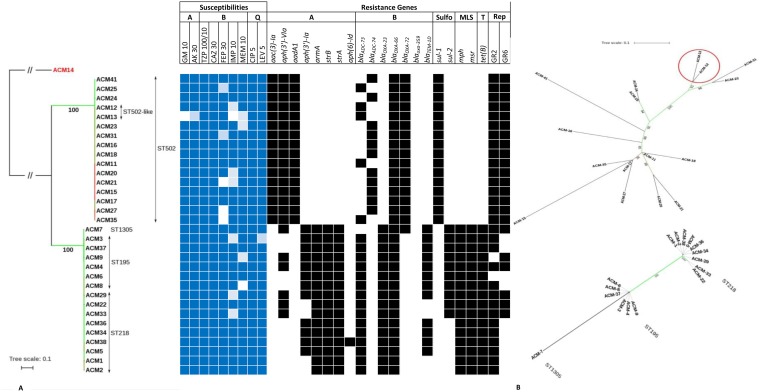
Maximum-likelihood phylogeny based on SNPs across A. baumannii isolates. (A) Branch lengths are proportional to the number of nucleotide substitutions per site. Bootstrap values are indicated by numbers below branches as well as by branch coloring (green, high confidence levels; red, low confidence levels). Dashed lines connect labels with particular leaf nodes in order to avoid labels to overlap the branches. Classes of antibiotics are given the following markings: A, aminoglycosides; B, β-lactams; Q, quinolones; T, tetracyclines; GM, gentamicin; AK, amikacin; TZP, piperacillin-tazobactam; CAZ, ceftazidime; FEP, cefepime; IMP, imipenem; MEM, meropenem; CIP, ciprofloxacin; LEV, levofloxacin. Dark blue indicates resistant, light blue indicates intermediate susceptibility, and blank indicates sensitive. (B) Unrooted phylograms inferred from SNP data from core genomes of strains belonging to the Tn*2006*-mediated clone and cluster pMAL-1-mediated clone, respectively. Red circle in the pMAL-1-mediated clone highlights ST-2059 isolates. Bootstrap values are indicated by numbers below branches as well as by branch coloring (green, high confidence levels; red, low confidence levels). Dashed lines connect labels with particular leaf nodes in order to avoid labels to overlap the branches.

Antibiotic susceptibility testing was performed on the 41 A. baumannii isolates, and 92.8% (*n* = 38) were found to be nonsusceptible to both gentamicin and amikacin. ACM-13 showed intermediate resistance to amikacin and was susceptible to gentamicin. Most of the isolates were quinolone resistant; 95% (*n* = 39) were resistant to ciprofloxacin, 92.8% (*n* = 38) were resistant to levofloxacin, and one isolate (ACM-3) showed intermediate resistance to levofloxacin. Additionally, 95% (*n* = 39) were resistant to ceftazidime, imipenem, meropenem, and piperacillin-tazobactam, and 82.9% (*n* = 34) were resistant to cefepime. All isolates were susceptible to colistin, as determined by the obtained MIC values. Based on the findings described above, 95% of the isolates were classified as being extensively drug resistant (XDR) (20) (Fig. 2).

The antibiotic susceptibility testing results were confirmed through *in silico* detection of resistant determinants. Four different aminoglycoside-inactivating enzymes and their variants were detected. Aminoglycoside acetyltransferase (AAC) *aac(3′)-Ia* and *ant(3″)-Ia*, belonging to the aminoglycoside nucleotidyltransferase (ANT) family, were found in 56% (*n* = 23) and 53.6% (*n* = 22) of the isolates, respectively. Five variants of the aminoglycoside phosphotransferase (APH) enzyme were also identified. The most common was *aph(3′)-VIa*, being detected in 73% (*n* = 30) of the isolates. *strA* and *strB* variants of the *aph* enzyme were detected in 46.3% (*n* = 19), *aph(3′)-Ia* in 34% (*n* = 14), *aph(6′)-Id* in 2.4% (*n* = 1), and *armA* in 46.3% (*n* = 19) of the isolates.

Acinetobacter-derived cephalosporinase genes *bla*_ADC-73_ and *bla*_ADC-74_ were identified in 62.5% (*n* = 25) and 37.5% (*n* = 15) of the isolates, respectively. IS*Aba1* was detected upstream of *bla*_ADC-73_ and *bla*_ADC-74_ in the opposite direction. All of the isolates carried *bla*_OXA-66_, while only ACM-14 was positive for *bla*_OXA-359._ Class A β-lactamase (*bla*_TEM-1D_), in addition to macrolide resistance determinants *mph*(E) and *msr*(E), were also found in 31.7% (*n* = 13) and 44% (*n* = 18) of the isolates, respectively.

### PFGE.

As we suspected an outbreak scenario, we first typed the isolates using PFGE. Isolates showing a difference of more than three bands were classified as belonging to different pulsotypes (21). Accordingly, 21 different pulsotypes were identified and were aligned with the STs but not with the source of isolation. The isolates, based on the PFGE output, appeared to be circulating in the different wards of the hospital. The first clade included ST-195, ST-218, ST-425, ST1305, and ST-502, while the second included ST-502 and ST-2059, a new ST, in addition to ST-218 and ST-425 (Fig. 1).

### MLST.

*In silico* MLST analysis, based on seven housekeeping genes (*cpn60*, *gdhB*, *gltA*, *gpi*, *gyrB*, *recA*, and *rpoD*) using the Oxford scheme, revealed the presence of eight different STs compared to only five STs identified using the Pasteur MLST scheme. Given that the Oxford MLST scheme was more capable at differentiating between closely related isolates, we adopted it for all further analysis. ST-502, CC92 (2-3-1-100-12-2-3) (ST-636, CC2 Pasteur scheme), which was the most common (44%; *n* = 18), and ST-502-like (ST-2059, a new ST), CC92 (ST-636, CC2 Pasteur scheme), were detected among the studied isolates. The difference between ST-502-like (ST-2059) and ST-502 is three point mutations in the *rpoD* gene, a G→T substitution at 117th base pair position, a G→T substitution at the 366th position, and a T→C substitution at the 504th position. ST-218, CC92 (2-3-1-102-3-2-3) (ST-2, CC2 Pasteur scheme), and ST-195, CC92 (2-3-1-96-3-2-3) (ST-2-like, CC2 Pasteur scheme), represented 27% (*n* = 11) and 17% (*n* = 7) of the isolates, respectively. Two singletons, ST-1305, CC208 (2-3-1-96-12-2-3; ACM-7) (ST-2-like, CC2 Pasteur scheme), and ST-425, CC92 (2-3-1-100-3-2-3; ACM-39) (ST-2-like, CC2 Pasteur scheme), were also among the detected ST types.

### Mobile genetic elements.

The A. baumannii PCR-based replicon typing (AB-PBRT) method was used to characterize the circulating plasmids. AB-PBRT categorizes A. baumannii plasmids into homology groups (GRs) based on the nucleotide homology of their respective replicase genes. AB-PBRT results showed that 21.9% (*n* = 9) of the isolates carried homology group GR2, 2.4% (*n* = 1) carried GR6, and 68.3% (*n* = 28) carried both GR1 and GR6, related to a wide range of plasmids (22).

### Tn*2006*-linked clone.

Nineteen CRAB (46.3%) isolated from 14 patients carried the *bla*_OXA-23_ carbapenemase gene in addition to intrinsically carrying *bla*_OXA-66_. These constituted the Tn*2006*-linked circulating clone in the outbreak. *bla*_OXA-23_ is usually associated with plasmids or integrated through transposons into the A. baumannii chromosome (10). These transposons are highly diverse and were designated Tn*2006*, Tn*2007*, Tn*2008*, and Tn*2009* (23). *bla*_OXA-23_ was carried by a chromosomally integrated Tn*2006*. The mobilization of Tn*2006*, being detected in ST-195, ST-218, and ST-1305, was facilitated by the IS*Aba1* bracketing of *bla*_OXA-23_. The remaining constituents of Tn*2006* were *yeeA* (encoding the putative DNA methylase), DEAD (encoding the putative Asp-Glu-Ala-Asp helicase), and ATPase (encoding the putative AAA ATPase) genes.

The integration site of Tn*2006* was the same among all the isolates of the Tn*2006*-linked clone. Tn*2006* was integrated inside a dienelactone hydrolase family protein, dividing it into two fragments, one 670 bp upstream of Tn*2006* and a smaller 145-bp fragment downstream of Tn*2006*.

Tn*2006* was associated with the AbaR25 resistance island, as has been previously described for Tn*2006* (23, 24). AbaR25 had the same genetic environment as AbaR25-I type (24).

The Tn*2006*-linked clone (ACM-2-6, ACM-8, ACM-9, ACM-34, ACM-36, and ACM-37) also harbored a circulating cryptic plasmid showing 100% BLAST sequence similarity to pA85-2 (GenBank accession number CP021786) and to pAB0057 (GenBank accession number CP001183) (8). The plasmid was also 99.99% similar to pAb-G7-1 (GenBank accession number KJ586856) (25), with only one base pair substitution difference (A→C). pA85-2 carried no resistance determinants but harbored a BrnT/BrnA toxin-antitoxin system, TonB-dependent receptor, and several hypothetical proteins, including septicolysin.

Interestingly, ACM-7 and ACM-28 carried both a chromosomal Tn*2006* mobilizing *bla*_OXA-23_ and a pMAL-1 plasmid carrying *bla*_OXA-72_, with no evidence of *bla*_OXA-72_ exchange between the plasmid and the chromosome (Table S2).

10.1128/mSphere.00934-19.2 TABLE S2 Plasmids identified based on contigs generated by SPAdes. Y, yes; N, no. Download Table S2, PDF file, 0.03 MB. Copyright © 2020 Makke et al. 2020 Makke et al. This content is distributed under the terms of the Creative Commons Attribution 4.0 International license.

### pMAL-1-linked clone.

**FIG 3:**
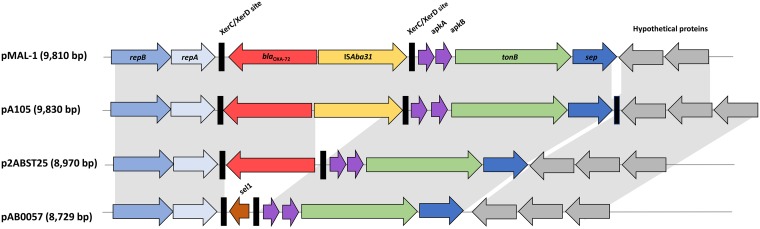
Comparative schematic representation of pMAL-1 and its closely related plasmids. pMAL-1 was aligned and compared with the three most closely related plasmids, pA105-2 (GenBank accession no. KR535993.1), p2ABST25 (GenBank accession no. AEPA01000396.1), and pAB0057 (GenBank accession no. NC_011585.1). Genes in pMAL-1 are annotated and colored with the following scheme: *repB*, blue; *repA*, light blue; XerC/XerD recombination sites, black; *bla*_OXA-72_, red; IS*Aba31*, yellow; *apkA-apkB*, purple; *tonB*, green; *sep*, dark blue; hypothetical proteins, gray.

Nineteen ST-502/2059 CRAB (48.7%) isolates were recovered from 15 patients and harbored the intrinsically carried *bla*_OXA-66_ in addition to *bla*_OXA-72_. Genome analysis of ACM-17, a representative of OXA-72-producing isolates, revealed that *bla*_OXA-72_ was carried on a small 9,810-bp plasmid that was identical to a previously identified GR2 plasmid in A. baumannii isolated from Serbia in 2016 and designated pMAL-1 (GenBank accession no. KX230793.1). Subsequently, BLAST analysis and genome alignments showed that the remaining ST-502 isolates also carried the same pMAL-1 plasmid. This plasmid was conjugative and had a genetic backbone identical to that of pMAL-1, being in the same order and orientation as *repAci1-repAci2* (replicase genes), two XerC/XerD sites (tyrosine recombinase sites) bracketing *bla*_OXA-72_, as well as IS*Aba31*, *tonB*, *sep* (septicolysin-encoding gene), and a few hypothetical proteins. Other plasmids were also closely related to the one detected in this study, including pA105-2 (GenBank accession number KR535993.1), isolated from A. baumannii in 2015, p2ABST25 (GenBank accession number AEPA01000396.1; negative for IS*Aba31*), and pAB0057 (GenBank accession number NC_011585.1; differing in the region flanking XerC/XerD; replaced by *bla*_OXA-72_ and IS*Aba31* in pMAL-1) (Fig. 3).

ACM-11, ACM-17, ACM-25, and ACM-35, which were part of the pMAL-1 clone, harbored other plasmids, such as pTG22653 (GenBank accession number CP039519.1) and ABAY04001 (GenBank accession number MK386680.1) (Table S2).

### pMAL-2 circulating plasmid.

In ACM-4, ACM-7, and ACM-29, representing the Tn*2006*-linked clone, and in ACM-12, ACM-15, ACM-25, ACM-31, ACM-35, and ACM-41, representing the pMAL-1-linked clone, we detected a common 70,499-bp plasmid. The plasmid was 100% identical to pAB-MAL-2 (GenBank accession number KX230794.1) (26) but had additional hypothetical proteins and a toxin-antitoxin (TA) *higAB* system. pAB-MAL-2 carried the tellurite resistance gene in addition to a Zeta toxin family protein, an ornithine cyclodeaminase, and many hypothetical proteins with unknown functions while being negative for antibiotic resistance determinants (Table S2 and Fig. S1 to S3).

10.1128/mSphere.00934-19.4 FIG S1 Neighbor-joining phylogenetic tree constructed based on NCBI BLAST *higA* hit result pairwise alignment. *higA* query is depicted in yellow. Download FIG S1, TIF file, 0.7 MB. Copyright © 2020 Makke et al. 2020 Makke et al. This content is distributed under the terms of the Creative Commons Attribution 4.0 International license.

In order to better characterize the *higAB* system found on pAB-MAL-2, we compared it with a recently described plasmid, pAB120, having the *higBA2*_Ab_ TA module (27). Alignment of the TA components revealed 60.3% and 58.8% similarity to *higB2* and *higA* of pAB120, respectively. The *higAB* system was preceded by IS*Aba125* (Fig. S1). BLAST analysis showed 100% identity to *higB2* of A. baumannii ACICU plasmid pACICU2 (GenBank accession number CP031382) and to the type II toxin-antitoxin system RelE/ParE in many other A. baumannii plasmids. *higA* was 100% identical to antitoxin *higA1*, linked to A. baumannii ACICU plasmid pACICU2 and to transcriptional regulators in other A. baumannii plasmids.

### Whole-genome SNP phylogenetic analysis.

In total, 71,864 SNP sites were identified. Isolates were separated into two major clusters, of which two representative genomes were chosen to be additionally sequenced using PacBio long-read sequencing technology, ACM-2 (representing the isolates carrying *bla*_OXA-23_ and forming the Tn*2006-*mediated clone) and ACM-17 (representing the isolates carrying *bla*_OXA-72_ and forming the pMAL-1-mediated clones). The results obtained confirmed the presence of two circulating clones. A total of 1,207 SNP sites were detected in the Tn*2006*-linked clone, while only 57 SNPs were detected in the pMAL-1-linked clone (Fig. 2).

### Outbreak analysis.

To reconstruct the suspected outbreak based on statistical analyses and core genome alignments, and taking into consideration collection dates, we used the R package Outbreaker (28). Outbreaker allows for a Bayesian reconstruction of disease outbreaks by combining both epidemiologic and genomic data. As a result, a bell-shaped distribution of the mutations per site and generation was observed in the case of the Tn*2006*-linked clone, along with a tight clustering of the isolates in an interconnected nodular network (Fig. S4). Clearly localized SNP-generating hot spots were favored to produce genomic diversity, as both recombination- and non-recombination-induced single-nucleotide variants almost always occurred in a few defined regions (SNP density ranging from 0.0003 to 0.0012). This included genes encoding diacylglycerol kinase, 3′,5′-cyclic-nucleotide phosphodiesterase, BapA prefix-like domain-containing protein, translation error-prone DNA polymerase V autoproteolytic subunit, and epoxyqueuosine reductase QueH. An exponential decrease of the infectivity of the cases based on the probability of infecting another patient up to 20 days after infection was observed (Fig. S5).

10.1128/mSphere.00934-19.5 FIG S2 Neighbor-joining phylogenetic tree constructed based on NCBI BLAST *higB* hit result pairwise alignment. *higB* query is depicted in yellow. Download FIG S2, TIF file, 0.8 MB. Copyright © 2020 Makke et al. 2020 Makke et al. This content is distributed under the terms of the Creative Commons Attribution 4.0 International license.

10.1128/mSphere.00934-19.6 FIG S3 Overview of plasmid pAB-MAL-2 harboring the *higAB* TA system. Hypothetical proteins are shown in grey, insertion sequences in yellow, and components of the TA systems in red. Download FIG S3, TIF file, 0.7 MB. Copyright © 2020 Makke et al. 2020 Makke et al. This content is distributed under the terms of the Creative Commons Attribution 4.0 International license.

10.1128/mSphere.00934-19.7 FIG S4 Bayesian transmission network analysis of the Tn*2006*-mediated clone. (A) Histogram showing the posterior distribution of the mutation rate. Mutations per site and generation were assessed based on their frequencies in the sampled cases. (B) Box-and-whisker plots showing the progression of estimated infection dates of the Tn*2006*-mediated clone. The cases are arranged based on how ancestral their core genomes are. (C) Inferred incidences plotted against estimated time of infection. The plot shows an incidence rate of 1 for almost all cases, indicating the progression of one outbreak throughout the 238 days. (D) Histogram showing the generation time distribution of the outbreak cases. Download FIG S4, TIF file, 0.9 MB. Copyright © 2020 Makke et al. 2020 Makke et al. This content is distributed under the terms of the Creative Commons Attribution 4.0 International license.

10.1128/mSphere.00934-19.8 FIG S5 SNP distances in the Tn*2006*-mediated clone. (A) Isolates belonging to the Tn*2006*-mediated clone and their relative SNP distances from the other isolates of the cluster. (B) Graph showing the density of the SNPs relative to their positions in the genomes of the sequenced isolates. A (780000 to 784000), CDS QCH35791.1, diacylglycerol kinase; CDS QCH35792.1, 3′,5′-cyclic-nucleotide phosphodiesterase; CDS QCH35793.1, BapA prefix-like domain-containing protein; B (2162000), CDS QCP38786.1, translesion error-prone DNA polymerase V autoproteolytic subunit; C (2280558), CDS QCP45253.1, epoxyqueuosine reductase QueH. Download FIG S5, TIF file, 1.3 MB. Copyright © 2020 Makke et al. 2020 Makke et al. This content is distributed under the terms of the Creative Commons Attribution 4.0 International license.

**FIG 4:**
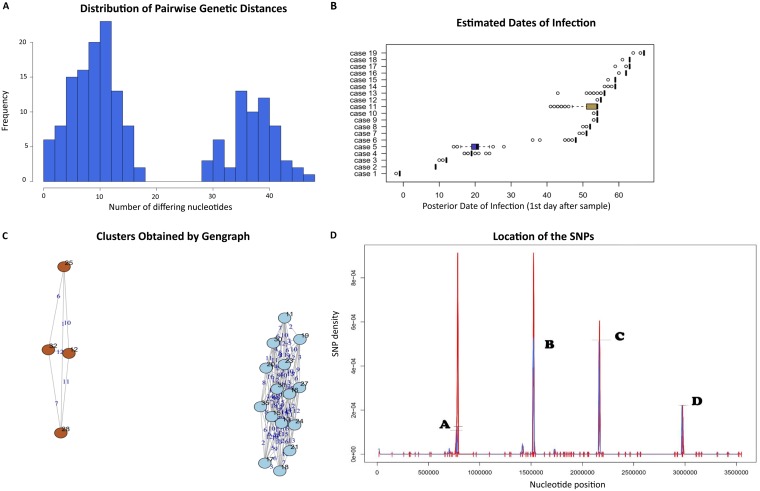
Bayesian transmission network analysis of the pMAL-1-mediated clone. (A) Histogram showing the distribution of pairwise genetic distances of the isolates and the relative frequencies of the genetic distances of the isolates of the pMAL-1-mediated clone. (B) Box-and-whisker plots of the estimated dates of infection. The cases are arranged based on how ancestral their core genomes are. (C) Isolates of the pMAL-1-mediated clone and their relative SNP distances from the other isolates of the cluster. At a hamming distance of 20, the isolates were arranged into two apparent subclones based on SNPs shared by some isolates in their core genomes introduced during the progression of the outbreak. (D) Graph showing the density of the SNPs relative to their positions in the genomes of the sequenced isolates: A (bp 784121), BapA prefix-like domain-containing protein; B (bp 1521949), DUF2750 domain-containing protein FDN01_10955; C (bp 2162660), stress-induced protein; D (bp 2971361), putative pilus assembly protein FilE.

On the other hand, in the pMAL-1-linked clone, the pairwise genetic distances had a clear bimodal distribution showing two bell-shaped curves (Fig. 4A). Based on this distribution, we speculated that the pMAL-1-linked clone encompassed two subclones. The mean mutation rate was 10, ranging from 0 to 18 for subclone I, while for subclone II we found 35 mutations, ranging between 28 and 45 (Fig. 4B). A high density of SNPs (ranging between 2 × 10^−4^ and 10 × 10^−4^) was observed within specific loci in the core genomes. These were genes encoding BapA prefix-like domain-containing protein, DUF2750 domain-containing protein, stress-induced protein, and putative pilus assembly protein FilE.

## DISCUSSION

In this study, a CRAB hospital outbreak caused by two distinct clones was tracked using PFGE, MLST, and WGS analysis over 9 months. Our analysis revealed a pMAL-1-linked clone, including ST-502 and ST-2059 isolates carrying *bla*_OXA-72_, along with two distinct subclones. A second Tn*2006*-linked clone was also identified, including ST-1305, ST-195, and ST-218 and carrying *bla*_OXA-23_. Both clones were circulating in different hospital wards and causing similar rates of mortality. WGS analysis was used to understand and elucidate the transmission pathways and to demonstrate the diversity of CRAB isolates and the associated mobile genetic elements.

The mobilization and spread of the chromosomal Tn*2006* transposon to three different STs (ST-195, ST-218, and ST-1305) was facilitated by the presence of two copies of IS*Aba1* upstream and downstream of *bla*_OXA-23_. Interestingly, out of the many transposons carrying the *bla*_OXA-23_ gene, only Tn*2006* has been shown to be highly mobile (29). *bla*_OXA-23_ was originally identified in Acinetobacter radioresistens, with *bla*_OXA-23_-mediated resistance to carbapenem being detected only in the presence of a strong promoter, such as IS*Aba1* (29). IS*Aba1*, which belongs to the IS*4* family of insertion sequence elements, provides a promoter motif mediating the increased expression of *bla*_OXA-23_ and is involved in facilitating its own mobilization not only to A. baumannii but also to Proteus mirabilis (10). Upon its first description in 1995 in A. baumannii, *bla*_OXA-23_ carried on plasmids or transposons became widespread and was detected in isolates recovered from the United Kingdom, France, Romania, Brazil, South Korea, United Arab Emirates, Egypt, and Iraq (10, 30, 31). *bla*_OXA-23_ was detected previously in GC2 CRAB isolates in Lebanon and was recognized as being the most common carbapenemase in the country (17, 30). The Tn*2006*-linked clone also carried a cryptic pA85-2 plasmid that appears to be commonly found in the GC1 lineage (32). The advantage conferred by this plasmid is still unknown.

The pMAL-1-linked clone included ST-502 and ST-2059 isolates and harbored the *bla*_OXA-72_ gene. This is the first report of ST-502 and ST-2059 A. baumannii in Lebanon. Carbapenem-resistant ST-502 isolates have been reported previously in Brazil (33), Bulgaria (34), and South Africa (35). *bla*_OXA-72_ was first identified in 2004 in A. baumannii isolated from Taiwan, and since then it was detected in a few other countries, including Brazil, France, and the United States (36). The pMAL-1 plasmid was very similar to p2ABST25, with the latter being negative for IS*Aba31* (26). The presence of IS*Aba31* upstream of *bla*_OXA-72_ has been reported previously in pMAL-1 (26), yet its effects on *bla*_OXA-72_ expression require further investigations. No previous reports showed the presence of p2ABST25-positive isolates in Lebanon or any of the neighboring countries, and as such pMAL-1 could have been introduced rather than evolved from another circulating plasmid.

We also revealed that the Tn*2006*-linked clone harbored a pA85-2 plasmid carrying persistence- and virulence-related genes, such as a BrnT/BrnA toxin-antitoxin system, a TonB-dependent receptor, and a septicolysin. The BrnT toxin-antitoxin system is required for RNA cleavage and control of bacteriostasis in Brucella abortus (37). TonB-dependent transporters are outer membrane proteins that bind and transport siderophores in addition to vitamin B12, nickel complexes, and carbohydrates (38). Septicolysin, on the other hand, is a pore-forming toxin with cytolytic activity that mediates invasion (39).

The pAB-MAL-2 plasmid was found circulating in both the Tn*2006*- and the pMAL-1-linked outbreak clones. pAB-MAL-2 detected in this study was slightly different from that in previous reports (26). The plasmid was additionally positive for a TA system showing 60% similarity to a recently described *higBA2*_Ab_ TA module on pAB120 (27). The *higAB* system found on the pAB-MAL-2 plasmid in our isolates was preceded by IS*Aba125.* IS*Aba125* was previously associated with the dissemination of *bla*_NDM-1_ (40). The presence of IS*Aba125*, a strong promoter, upstream of the TA system (41) is an important finding. TA systems are genetic loci involved in plasmid maintenance and in regulating bacterial stress responses linked to pathogen virulence and formation of drug-resistant persister cells and biofilms (42). The *higBA2* gene detected in this study is a reverse TA, where the toxin gene is the first in the operon. HigB2 functions as an RNase capable of conferring maintenance of unstable plasmid in Acinetobacter, and it is neutralized with HigA2 antitoxin (27).

A total of 1,207 SNP sites were detected within the Tn*2006*-linked clone harboring *bla*_OXA-23_, compared to only 57 SNP sites identified within the pMAL-1-linked clone harboring *bla*_OXA-72_. Bayesian analysis revealed the transmission events with a high degree of certainty. During the outbreak, the isolates underwent genomic remodeling at clearly defined loci. Recombination events, as well as increased variance in the nucleotide sequences within a few genes, served as the primary drivers of diversity. In particular, and in both clones, a high density of SNPs was observed in BapA, a biofilm-associated protein mediating biofilm formation and adhesion to host cells (43). In the Tn*2006*-linked clone, one of the genes that accumulated a large number of SNPs encoded a diacylglycerol kinase, a small integral membrane protein (44). Diacylglycerol kinase was previously shown to be upregulated in colistin-resistant A. baumannii and was hypothesized to increase the integrity of the cell membrane, favoring drug resistance (45).

In conclusion, by following the pattern of transmission and based on the molecular epidemiology and genomics data, we confirm the polyclonal nature of the outbreak. Using PFGE, MLST, and PCR typing methods in combination with WGS data and SNP-based Bayesian transmission network analysis, we were able to characterize at high-scale resolution a hospital CRAB outbreak caused by a Tn*2006*-linked clone carrying *bla*_OXA-23_ and a pMAL-1-linked clone carrying *bla*_OXA-72_. An outbreak driven by chromosomally encoded resistance determinants had a more consistent evolution and was easier to retrace using core genome sequence data from short genome reads. Alternatively, a plasmid-driven outbreak followed less of a straight evolutionary path during the sampling. Both clones additionally harbored circulating plasmids, ensuring the survival of the fittest and contributing to virulence.

## MATERIALS AND METHODS

### Ethical approval.

Ethical approval was not required, as the isolates were collected as part of routine clinical care and patient data collection followed patient discharge from the hospital and/or death. No additional isolates were collected beyond those obtained from routine clinical care, and no diagnostic or treatment decisions were affected by the outcomes of this study.

### Bacterial isolates.

A total of 41 A. baumannii isolates were collected between April and December 2016 from hospitalized patients at a 544-bed hospital in Lebanon and were designated ACM-1 to -37 based on the date of their isolation and ACM-38 to -41 for the isolates with no trackable records. The initial identification of Acinetobacter species was carried out using automated microbial identification systems (Vitek and BD Phoenix).

### Antimicrobial susceptibility testing.

Antimicrobial susceptibility testing was performed using the disk diffusion method on Mueller-Hinton agar to determine resistance patterns against nine different antibiotics: gentamicin, amikacin, piperacillin-tazobactam, ceftazidime, cefepime, imipenem, meropenem, ciprofloxacin, and levofloxacin. The results were interpreted according to Clinical and Laboratory Standards Institute (CLSI) guidelines (M100-S29) (46). Isolates were considered extensively drug resistant (XDR) if they were nonsusceptible to at least one agent in all but two or fewer antimicrobial classes (20).

MICs were determined using the Etest (bioMérieux, France) method for gentamicin, cefepime, ciprofloxacin, trimethoprim-sulfamethoxazole, amikacin, piperacillin-tazobactam, and colistin and the broth microdilution method for colistin, meropenem, and imipenem (Sigma-Aldrich, USA). For the Etest, bacterial suspensions with turbidity equivalent to 0.5 McFarland standard were prepared and spread onto Mueller-Hinton agar (MHA) (Bio-Rad Laboratories, Inc., USA), followed by the addition of Etest strips. The plates then were incubated at 37°C for 24 h. For the broth microdilution method, cation-adjusted Mueller-Hinton broth (Sigma-Aldrich, USA) was used according to CLSI recommendations. Serial dilutions of each of the three antibiotics (32 to 0.062 μg/ml) were prepared. A. baumannii isolates were suspended to 0.5 McFarland turbidity and added to 96-well microdilution plates. Microplates were incubated at 35°C for 20 to 24 h. The lowest antimicrobial drug concentration at which there was no growth was considered the MIC.

### PFGE.

Genomic DNA plugs of A. baumannii were prepared according to the protocol developed by Seifert et al. (47). Briefly, plugs were digested using ApaI (Thermo Fisher Scientific, MA, USA) for 2 h at 37°C. DNA fragments were then separated on 1% Seakem Gold gel using a CHEF DR-III system (Bio-Rad Laboratories, Inc., CA, USA) for 16 h with initial and final switch time of 7 s and 20 s, respectively. To compare the patterns on different gels, Salmonella enterica subsp. *enterica* serovar Braenderup (ATCC BAA664TM) was used as the reference for band size using XbaI restriction digestion (Thermo Fisher Scientific, MA, USA). The gel then was stained with ethidium bromide and viewed under UV light. Fingerprints were analyzed using BioNumerics software, version 7.6.1 (Applied Maths, St-Martens-Latem, Belgium). Bands not detected automatically were manually assigned. Fingerprints were clustered according to the band-based coefficient, which measures similarity based upon common and different bands with 0.5% optimization and 0.5% tolerance of band matching.

### Conjugation.

Conjugation experiments were performed as described previously (48) using isolate ACM-17 of the pMAL-1-linked clone and carrying *bla*_OXA-72_ as a donor and Escherichia coli J53, resistant to sodium azide, as a recipient. ACM-17 and E. coli J53 were mixed at a ratio of 4:1 (donor to recipient) in Luria-Bertani broth, and the mixture was incubated at 37°C for 1 h. To select for transconjugants, serial dilutions of the cultures were plated on UriSelect agar containing imipenem (4 μg/ml) and sodium azide (100 μg/ml).

### Whole-genome sequencing.

DNA extraction was performed using the NucleoSpin tissue kit (Macherey-Nagel, Germany) according to the manufacturer’s instructions. Library preparation was done using the Nextera XT DNA library preparation kit (Illumina). Sequencing of the library was done on an Illumina MiSeq using a paired-end 500-cycle protocol with a read length of 250 bp. FastQC, version 1.0.0 (49), was used for quality control. Reads were trimmed by the Trimmomatic tool, v0.36 (50). Paired-end reads were assembled using SPAdes, v3.12.0 (51), and annotated using the RAST server (http://rast.nmpdr.org) (52).

Two isolates (ACM-2 and ACM-17) were additionally sequenced using PacBio long-read sequencing technology on the Sequel platform (Pacific Biosciences, CA, USA). ACM-2 represented the Tn*2006*-mediated clone, and ACM-17 represented the pMAL-1-mediated clone. Library preparation was done according to the manufacturer’s instructions for microbial multiplexing. G-tubes (Covaris, USA) were used for DNA shearing, and no size selection was performed. Resulting chromosomal contigs of ACM-2 and ACM-17 were corrected by Illumina data with Pilon, v1.23 (53), and the overlapping ends of chromosomes were trimmed after manual inspection of reads mapped by BWA-MEM algorithm as implemented in BWA, v0.7.17 (54), and Bowtie, v2.3.4.2 (55). Genome assembly was done using HGAP4 (56) with minimum seed coverage of 30. PCR-based amplification was used to fill the sequence gaps.

### Genome analysis.

Resfinder (57), MLST 1.8 server, ISfinder database (58), BLAST (59), and the open reading frame (ORF) finder tool (59) were utilized to identify resistance genes, sequence types (STs), ISs, and plasmid identification and annotation, respectively. Mauve (version 2.3.1) was used for comparative genome alignments (60). The core genome alignments of each clone were used for outbreak analysis. Ape was used to infer basic phylogeny of the aligned core genomes (61). Adegenet was used to cluster genetic fragments that were found to be identical between the isolates from each outbreak and to perform a multivariate analysis of the genetic markers using the SeqTrack algorithm (62); this was used to visualize the genetic relatedness of the isolates spatially. plasmidSPAdes, version 3.10.1 (63), was employed to generate separate plasmid contigs, and the output was visualized through Bandage and an assembly graph viewer (64).

We used Outbreaker (28) to help elucidate the circulating clones that were directly linked to the studied outbreak. Outbreaker is based on collection dates and a reconstruction of the outbreak based on the evolution of core genome SNPs outside identified recombination spots in all studied genomes.

To detect recombinant regions, core genomes were aligned using Gubbins (65). Recombinations were masked using maskrc (https://github.com/kwongj/maskrc-svg).

### Phylogenetic analysis.

For the purposes of phylogenetic analysis of all the recovered isolates, along with ACM-14, which was used as an outgroup, core genome sequences were extracted from the contigs using an in-house script. The script was used to extract all nonrepetitive homologous sequences that are longer than 399 bp using NUCmer, version 3.1 (66), output as generated by QUAST, version 5.0.0 (67). Core genome sequences were aligned by MAFFT, version 7.407 (68), using default gap penalties and the memsave parameter, FFT-NS-2 strategy, and iterative refinement with a maximum of two iterations. SNP sites then were extracted from aligned sequences using the SNP-sites tool, version 2.4.0 (69).

In order to infer the phylogeny of the two clones, quality-trimmed Illumina reads were mapped to the chromosomal sequences (ACM-2 or ACM-17) using Bowtie, version 2.3.4.2 (55). SNPs were consequently called using VarScan, version 2.4.3 (70), with minimum read depth set to 8, minimum base quality of 20, and variant allele frequency of ≥0.8. All sites where at least one isolate had a read depth of <8 were removed from the final data sets.

All three data sets were analyzed by jModelTest, version 2.1.10, to determine the most appropriate models of nucleotide substitution (71). Phylogenetic analyses were performed by RAxML, version 8.2.10 (72), and robustness of the inferred topologies was assessed by 500-bootstrap replicate analyses. Topologies of the trees were visualized using iTOL, version 4.3.2 (73), and edited by Inkscape, version 0.92 (www.inkscape.org), and Gimp, version 2.10.6 (www.gimp.org).

### Plasmid analysis.

Sequence gaps resulting from short Illumina reads were closed using PCR and Sanger sequencing. Seven primer pairs were designed using SeqBuilder software (Lasergene, Madison, WI). For sequence analysis and annotation, the BLAST algorithm (www.ncbi.nlm.nih.gov/BLAST), the ISfinder database (www-is.biotoul.fr/), and the ORF finder tool (www.bioinformatics.org/sms/) were utilized. Comparative genome alignments were performed using Mauve, v2.3.1 (60).

### Plasmid typing.

The PCR-based replicon typing method for A. baumannii developed by Bertini et al. (22) was used to determine the plasmid content of the 41 isolates. With this method, the 27 known A. baumannii replicase (rep) genes are grouped into 19 homology groups according to their nucleotide sequence similarities. Six multiplex PCRs were done using primers designed by Bertini et al. (22).

### Accession number(s).

The draft genomes were deposited in the NCBI databases under the accession numbers listed in Table S3 in the supplemental material.

10.1128/mSphere.00934-19.3 TABLE S3 Genome information and accession numbers. Nb, number. Download Table S3, PDF file, 0.02 MB. Copyright © 2020 Makke et al. 2020 Makke et al. This content is distributed under the terms of the Creative Commons Attribution 4.0 International license.
