# Supplementary material for: Whole-Genome-Sequence-Based Characterization of Extensively Drug-Resistant Acinetobacter baumannii Hospital Outbreak
Source: mSphere. 2020 Jan 15;5(1):e00934-19. doi: 10.1128/mSphere.00934-19 (PMC6968657; doi:10.1128/mSphere.00934-19)
Supplement: TABLE S1 [file mSphere.00934-19-st001.pdf]

Patient	Sample	Source	Sex	Age Range	Outcome	Underlying Disease	Date of specimen	GM MIC	GM 10	AK MIC	AK 30	TZP MIC	TZP 100/10	CAZ 30	FEP MIC	FEP 30	IMP MIC	IMP 10	MEM MIC	MEM 10	CIP MIC	CIP 5	LEV 5	COL MIC	COL 50	TMP-SMX MIC	TMP-SMX
**1**	**ACM-1**	DTA	F	0-1	Survived	Apnea	April-2016	8		≥256		≥256			≥128		8		8		≥32			0.94		1.5	
**2**	**ACM-2**	Eye Culture	F	0-1	Survived	Premature, 2nd twin, respiratory distress	May-2016	8		≥256		≥256			128		8		8		≥32			1		1.5	
**3**	**ACM-3**	DTA	F	80-89	Died	Head trauma, multiple comorbidities	May-2016	4		≥256		≥256			128		4		8		≥32			0.09		≥32	
**4**	**ACM-4**	BLOOD	F	60-69	Died	Chemotherapy	June-2016	≥256		≥256		≥256			≥128		8		8		≥32			0.75		≥32	
**5**	**ACM-5**	DTA	M	60-69	Died	Head trauma, septicemia	June-2016	8		≥256		≥256			128		8		8		≥32			1		≥32	
**6**	**ACM-6**	BW	F	50-59	Died	Multiple comorbidities, diabetes, foot amputation, sepsis	June-2016	≥256		≥256		≥256			≥128		8		8		≥32			1		≥32	
	**ACM-8**	DTA					June-2016	≥256		≥256		≥256			128		8		0.5		≥32			0.75		≥32	
**7**	**ACM-7**	Surgical (abdm)	F	50-59	Died	Acute myeloid leukemia, ulcer	June-2016	12		≥256		≥256			≥128		8		8		≥32			0.38		≥32	
**8**	**ACM-9**	DTA	F	40-49	Survived	LOC, intubated, fever, dysuria	June-2016	8		≥256		≥256			≥128		8		4		≥32			0.13		≥32	
**9**	**ACM-10**	BLOOD	F	40-49	Died	Cardiac arrest, brain anoxia injury, sepsis	July-2016	0.19		0.5		≥256			1.5		0.5		1		0.25			0.13		0.25	
**10**	**ACM-11**	DTA	F	60-69	Died	Multiple comorbidities, hyponatremia, hyperkalimia(kidney injury)	July-2016	8		32		≥256			24		>8		>8		≥32			0.13		≥32	
	**ACM-18**	BW					September-2016	8		96		≥256			≥128		8		8		≥32			0.06		≥32	
**11**	**ACM-12**	BW	M	40-49	Survived	Respiratory distress, fever, lung pneumonia	August-2016	2		64		≥256			128		4		8		≥32			0.38		≥32	
**12**	**ACM-13**	DTA	M	60-69	Died	Jaundice	August-2016	1.5		32		≥256			32		2		4		≥32			0.5		≥32	
**13**	**ACM-14**	DTA	F	70-79	Died	Decreased LOC, dysarthria, severe respiratory distress	August-2016	0.25		20		0.02			2		0.25		4		0.19			0.13		0.19	
**14**	**ACM-15**	DTA	F	50-59	Died	Lung adenocarcinoma	September-2016	16		64		≥256			48		8		8		≥32			0.13		≥32	
**15**	**ACM-17**	DTA	F	50-59	Survived	Decreased level of consciousness, fever, cough	September-2016	16		96		≥256			64		8		8		≥32			0.38		≥32	
	**ACM-19**	Sputum					September-2016	12		48		≥256			128		8		8		≥32			0.02		≥32	
**16**	**ACM-20**	Surgical	F	60-69	Survived	Malignant malenoma of skin	September-2016	12		32		≥256			64		4		8		≥32			0.02		≥32	
**17**	**ACM-21**	Surgical	M	80-89	Died	Hyperplasia of prostate, diabetes, septic shock	September-2016	16		≥256		≥256			128		4		8		≥32			0.75		≥32	
	**ACM-31**	DTA					October-2016	4		64		≥256			24		≥32		8		≥32			0.25		≥32	
	**ACM-35**	Surgical					October-2016	16		≥256		≥256			96		≥32		8		≥32			0.13		≥32	
**18**	**ACM-16**	DTA	F	70-79	Died	Meningioma	September-2016	16		32		≥256			≥128		≥32		8		≥32			0.19		≥32	
**19**	**ACM-22**	BLOOD	M	2-9	Survived	Deteriorating edema anuria, sepsis, seizures, pneumonia, surgery (peritoneal catheter insertion)	September-2016	≥256		≥256		≥256			≥128		≥32		16		≥32			0.38		≥32	
	**ACM-24**	P. FLUID					September-2016	≥256		≥256		≥256			≥128		≥32		8		≥32			0.75		≥32	
	**ACM-25**	BLOOD					September-2016	6		48		≥256			≥128		≥32		8		≥32			1		≥32	
	**ACM-26**	P Fluid					September-2016	≥256		≥256		0.16			≥128		≥32		8		≥32			0.13		≥32	
	**ACM-28**	BLOOD					September-2016	4		64		≥256			48		≥32		8		≥32			0.75		≥32	
	**ACM-29**	Peritoneal					September-2016	≥256		≥256		≥256			32		≥32		8		≥32			0.25		≥32	
	**ACM-30**	Surgical					September-2016	3		128		≥256			≥128		≥32		16		≥32			0.38		≥32	
	**ACM-33**	Peritoneal					October-2016	≥256		≥256		≥256			64		≥32		8		≥32			0.38		≥32	
**20**	**ACM-23**	DTA	M	60-69	Survived	Dyspnea, decreased levels of consciousness, respiratory distress	October-2016	2		16		≥256			48		≥32		4		≥32			0.75		≥32	
	**ACM-32**	DTA					September-2016	12		256		≥256			32		≥32		4		≥32			0.5		≥32	
**21**	**ACM-27**	DTA	M	60-69	Died	Respiratory distress, palsy, septic shock	September-2016	16		64		≥256			≥128		≥32		8		≥32			0.05		≥32	
**22**	**ACM-34**	BLOOD	F	0-1	Survived	Premature, bacterial sepsis	October-2016	≥256		≥256		≥256			≥128		≥32		8		≥32			0.5		4	
	**ACM-36**	surgical (Umbl)					October-2016	≥256		≥256		≥256			≥128		≥32		8		≥32			0.13		1.5	
**23**	**ACM-37**	DTA	M	90-99	Died	Hyperplasia of prostate, bacterial pneumonia, septic shock, atrioventricular block	December-2016	≥256		≥256		≥256			128		≥32		8		≥32			0.19		≥32	
**24**	**ACM-38**	-	-	-	-	-	-	1		≥256		≥256			46		≥32		8		≥32			0.5		≥32	
**25**	**ACM-39**	-	-	-	-	-	-	12		64		≥256			48		≥32		8		≥32			0.13		≥32	
**26**	**ACM-40**	-	M	50-59	-	-	-	≥256		≥256		≥256			≥128		≥32		8		≥32			0.13		≥32	
**27**	**ACM-41**	-	-	-	-	-	-	24		96		≥256			≥128		≥32		8		≥32			0.13		≥32	
