# Supplementary material for: Whole-Genome-Sequence-Based Characterization of Extensively Drug-Resistant Acinetobacter baumannii Hospital Outbreak
Source: mSphere. 2020 Jan 15;5(1):e00934-19. doi: 10.1128/mSphere.00934-19 (PMC6968657; doi:10.1128/mSphere.00934-19)
Supplement: TABLE S2 [file mSphere.00934-19-st002.pdf]

Sample	Plasmid	Accession#	% Identity	*bla* $_{OXA-72}$	*bla* $_{OXA-23}$
ACM-1	ABAY15001	MK386684.1	99	N	N
ACM-1	AB34299	CP014293.1	99	N	N
ACM-2	pA85-2	CP021786.1	100	N	N
ACM-3	pA85-2	CP021786.1	100	N	N
ACM-4	pMAL-2	KX230794.1	100	N	N
ACM-4	pA85-2	CP021786.1	100	N	N
ACM-5	pA85-2	CP021786.1	100	N	N
ACM-6	pA85-2	CP021786.1	100	N	N
ACM-7	pMAL-2	KX230794.1	100	N	N
ACM-7	pMAL-1	KX230793.1	100	Y	N
ACM-8	pA85-2	CP021786.1	100	N	N
ACM-9	pA85-2	CP021786.1	100	N	N
ACM-11	pTG22653	CP039519.1	99	N	N
ACM-11	pMAL-1	KX230793.1	100	Y	N
ACM-12	pMAL-1	KX230793.1	100	Y	N
ACM-12	pMAL-2	KX230794.1	100	N	N
ACM-13	pMAL-1	KX230793.1	100	Y	N
ACM-15	pMAL-2	KX230794.1	100	N	N
ACM-15	pMAL-1	KX230793.1	100	Y	N
ACM-16	pMAL-1	KX230793.1	100	Y	N
ACM-17	pTG22653	CP039519.1	99	N	N
ACM-17	pMAL-1	KX230793.1	100	Y	N
ACM-18	pMAL-1	KX230793.1	100	Y	N
ACM-20	pMAL-1	KX230793.1	100	Y	N
ACM-21	pMAL-1	KX230793.1	100	Y	N
ACM-22	AB34299	CP014293.1	99	N	N
ACM-23	pMAL-1	KX230793.1	100	Y	N
ACM-24	pMAL-1	KX230793.1	100	Y	N
ACM-25	pMAL-1	KX230793.1	100	Y	N
ACM-25	pTG22653	CP039519.1	99	N	N
ACM-25	pMAL-2	KX230794.1	100	N	N
ACM-27	pMAL-1	KX230793.1	100	Y	N
ACM-29	AB34299	CP014293.1	99	N	N
ACM-29	pMAL-2	KX230794.1	100	N	N
ACM-31	pMAL-2	KX230794.1	100	N	N
ACM-31	pMAL-1	KX230793.1	100	Y	N
ACM-33	AB34299	CP014293.1	99	N	N
ACM-34	pA85-2	CP021786.1	100	N	N
ACM-35	ABAY04001	MK386680.1	99	N	N
ACM-35	pMAL-2	KX230794.1	100	N	N
ACM-36	pA85-2	CP021786.1	100	N	N
ACM-37	pA85-2	CP021786.1	100	N	N
ACM-38	ABAY15001	MK386684.1	100	N	N
ACM-38	AB34299	CP014293.1	99	N	N
ACM-41	pMAL-2	KX230794.1	100	N	N
ACM-41	pMAL-1	KX230793.1	100	Y	N
