# Supplementary material for: Whole-Genome-Sequence-Based Characterization of Extensively Drug-Resistant Acinetobacter baumannii Hospital Outbreak
Source: mSphere. 2020 Jan 15;5(1):e00934-19. doi: 10.1128/mSphere.00934-19 (PMC6968657; doi:10.1128/mSphere.00934-19)
Supplement: TABLE S3 [file mSphere.00934-19-st003.pdf]

ACM Label	Accession Numbers	Nb of contigs	N50	Coverage
ACM-1	MJAY00000000	108	165,005	68
ACM-2	MJAZ00000000	76	161,735	72
ACM-3	MJBB00000000	107	162,580	151
ACM-4	MJBA00000000	93	168,008	88
ACM-5	PYCX00000000	107	165,005	30
ACM-6	MJBC00000000	84	168,008	62
ACM-7	MJBD00000000	132	162,580	74
ACM-8	MJBE00000000	103	162,244	100
ACM-9	PYCV00000000	126	162,580	30
ACM-11	PYCW00000000	111	133,397	30
ACM-12	PYCY00000000	135	121,813	30
ACM-13	PYCZ00000000	187	132,022	30
ACM-14	PYDA00000000	102	121,152	30
ACM-15	PYDB00000000	124	133,397	30
ACM-16	PYDC00000000	118	132,002	30
ACM-17	PYDD00000000	124	124,392	30
ACM-18	PYDE00000000	120	132,002	30
ACM-19	PYDF00000000	132	106,813	30
ACM-20	PYDG00000000	220	118,630	30
ACM-21	PYDH00000000	112	118,753	30
ACM-22	PYDI00000000	126	168,008	30
ACM-23	PYDJ00000000	126	118,871	30
ACM-24	PYDK00000000	113	110,190	30
ACM-25	PXYP00000000	126	106,424	30
ACM-26	PYDL00000000	1,926	8,266	30
ACM-27	PYDM00000000	142	131,998	30
ACM-28	PYDN00000000	124	107,062	30
ACM-29	PYDO00000000	187	143,070	30
ACM-30	PYDP00000000	118	111,165	30
ACM-31	PYDQ00000000	136	132,002	30
ACM-32	PYDR00000000	136	107,062	30
ACM-33	PYDS00000000	102	165,005	30
ACM-34	QAGN00000000	102	165,005	30
ACM-35	QEHN00000000	179	111,165	30
ACM-36	QAGO00000000	99	165,005	30
ACM-37	QAGP00000000	123	162,244	30
ACM-38	QAGQ00000000	91	194,416	30
ACM-39	QAGR00000000	315	84,717	30
ACM-40	QAGS00000000	160	147,706	30
ACM-41	QAGT00000000	99	134,794	30
